# Population-Based, Spatial Analysis of Specialised Ambulatory Palliative Care in Mecklenburg-Western Pomerania, Germany, on the Basis of Reimbursement Data

**DOI:** 10.3390/ijerph20032231

**Published:** 2023-01-26

**Authors:** Maren Leiz, Kilson Moon, Laura Kerstin Rehner, Ulrike Stentzel, Franziska Radicke, Wolfgang Hoffmann, Neeltje van den Berg

**Affiliations:** 1 Institute for Community Medicine, University Medicine, 17475 Greifswald, Germany; 2 Institute for Nursing Science and Interprofessional Learning, University Medicine, 17475 Greifswald, Germany

**Keywords:** palliative care, claims data, regional differences, spatial accessibility, distribution, rural

## Abstract

In rural areas, healthcare providers, patients and relatives have to cover long distances. For specialised ambulatory palliative care (SAPV), a supply radius of max. 30 km is recommended. The aim of this study was to analyse whether there are regional disparities in the supply of SAPV and whether it is associated with the distance between the SAPV team’s site and the patient’s location. Therefore, anonymised data of the Association of Statutory Health Insurance Physicians of the Federal State of Mecklenburg-Western Pomerania (M-V) were retrospectively analysed for the period of 2014–2017. Identification as a palliative patient was based on palliative-specific items from the ambulatory reimbursement catalogue. In total, 6940 SAPV patients were identified; thereof, 48.9% female. The mean age was 73.3 years. For 28.3% of the identified SAPV patients (*n* = 1961), the SAPV teams had a travel distance of >30 km. With increasing distance, the average number of treatment days per patient increased. It was found that there are regional disparities in the provision of SAPV services in M-V and that local structures have an important impact on regional supply patterns. The distance between the SAPV team’s site and the patient’s location is not the only determining factor; other causes must be considered.

## 1. Introduction

In Germany, about 850,000 people die every year. According to the German Society for Palliative Medicine, up to 90% of these require some kind of palliative care [1]. Palliative care is the active and comprehensive care of a patient whose disease does not respond to curative treatment. The primary objective is to alleviate pain and other symptoms as well as social, psychological and spiritual problems [2,3]. Regardless of age, diagnosis and prognosis, every patient should have access to palliative care. This can only be ensured by a well-connected, multi-professional supply, which is particularly essential in peripheral and rural regions [3,4,5]. Comprehensive palliative care takes place at different levels: general and specialised ambulatory and inpatient care, and ambulatory volunteer hospice services [4,6]. Approximately 10% of the palliative care patients need specialised ambulatory palliative care (SAPV) [7].

SAPV offers healthcare services for patients who cannot be adequately cared for by other palliative services due to the complexity of their symptoms and medical condition. Despite the high level of care required, SAPV enables care in the patient’s familiar environment (e.g., at home or in a hospice) and hospitalisation can be avoided [7].

SAPV has to be ordered by a physician and approved by the statutory health insurance fund; the patient must also agree to the care. SAPV teams have their own contracts with the Associations of Statutory Health Insurance Physicians or health insurance funds in the respective federal states [7]. SAPV can be provided as a consulting service, coordination service, additive supportive care or complete patient care. The SAPV team works together with healthcare service providers of different professional groups (e.g., general practitioners, specialist physicians, nursing services, hospice services). Cooperation agreements between the SAPV team and its cooperation partners are the basis for cooperation within a region. Moreover, family caregivers provide substantial care for patients [5,8,9].

Palliative care will become more relevant in the future due to current demographic trends with an ageing population and a resulting increase in the demand for palliative care [3]. According to calculations by the German Federal Statistical Office, the proportion of people ≥65 years in the population will increase from 21.5% in 2018 to 30.9% in 2060 [10]. With increasing age, not only the individual risk of illness increases, but also the risk of multimorbidity [11,12,13,14].

Due to a low population density, rural areas are generally characterised by a lower density of medical facilities [15]. Especially in rural areas, healthcare providers, patients and relatives have to cover large areas and manage long distances [16,17,18]. The German Society for Palliative Medicine and the German Hospice and Palliative Association recommend that a 30 km supply radius for SAPV teams should only be exceeded in sparsely populated areas or in exceptional cases [19]. In Mecklenburg-Western Pomerania (M-V), Germany’s most sparsely populated federal state with a mean of 69 inhabitants/km^2^ [20], the 30 km recommendation cannot always be met. SAPV teams often have to cover large distances to reach their patients. As home visits are an important part of SAPV, long distances can be a barrier for this outpatient care.

The objective of this study is to analyse the regional supply by SAPV teams in order to meet the challenges in palliative care in rural regions, using the example of M-V. The focus of the analysis is on regions that are outside a 30 km radius of the SAPV team’s site. The research questions are:

1. Are there regional disparities in the utilisation of SAPV services?
2. Is the distance between the SAPV team’s site and the patient’s location associated with the utilisation of SAPV services?

## 2. Materials and Methods

### 2.1. Data

Analyses were performed using reimbursement data from the Association of Statutory Health Insurance Physicians in M-V. Patient individual data were available from January 2014 to December 2017. The data set contained reimbursed healthcare services, reimbursement quarter and date, practice anonym and zip code, patient anonym and zip code, patient date of birth and gender. Data from 10 of 12 SAPV teams in M-V were available. Of the two missing SAPV teams, one has separate contracts with the health insurance companies without involvement of the Association of Statutory Health Insurance Physicians M-V. One team did not agree to the use of their data for this analysis. The paediatric SAPV team was excluded from this analysis.

### 2.2. Patient’s Selection and Identification

The identification as a palliative care patient was based on the reimbursement of any palliative care-specific service in the reimbursement catalogue for ambulant healthcare (Table 1). The analyses included patients who received an initial palliative assessment between 2014 and 2017. If more than one initial assessment was performed, only the last one was considered. Additionally, only patients with residential zip codes within M-V were included. Since surcharges are granted for patients living in areas >30–50 km and >50 km away from the treating SAPV team’s site, distance-specific analyses could be performed according to these categories. When at least one service with distance surcharge was reimbursed, it was assumed that patients lived >30–50 km or >50 km from the SAPV team’s site, respectively. If patients living >30 km from the SAPV team’s site received a home visit for which no distance surcharge was charged, it was presumed that a cooperating physician or a cooperating nursing service in the nearby region of the patient treated this patient. Up to a distance of 30 km, no distinction can be made between services by an SAPV team or cooperation partners.

### 2.3. Data Analyses

Calculated were numbers of patients, total treatment days and treatment days per patient. The variables were shown as mean and 95% confidence interval (CI). Differences between groups are considered to be significant if CIs do not overlap. A bivariate choropleth map [21] shows the treatment days per patient related to the service areas. Bivariate choropleth maps show relationships between two variables. For this purpose, service areas were classified in three categories (≤30 km, >30–50 km, >50 km). The number of treatment days per patient was categorised through tertiles (>0–26 days, >26–36 days, >36 days). Additionally, a spatial autocorrelation (Global Moran’s I, Anselin Local Moran’s I) was conducted to analyse the autocorrelation within the number of treatment days per patient across space. The Global Moran’s I Index attains a value between −1 (perfect dispersion) and +1 (perfect clustering), with a z-score as standard deviation and *p*-value [22]. If the Global Moran’s I results in clustering, Local Moran’s I identifies concentrations of high values (“high-high cluster”), low values (“low-low cluster”) and spatial outliers (“high-low cluster” and vice versa) [23]. As a result, significant differences and similarities between the zip code areas can be revealed.

The address data of the SAPV teams’ sites were determined via the online physician information register in M-V [24]. The sites were geocoded using a geographic information system (GIS; ESRI^®^ArcGIS™ 10.7.1 Esri Inc., Redlands, CA, USA). The Central Information Register of Mecklenburg-Western Pomerania provided the daily updated (10.01.2018) number of inhabitants in five-year age groups according to districts. To fit to the palliative service data, the districts were transformed into zip code areas using the GIS. The number of inhabitants in five-year age groups were joined via the zip code to calculate the relative numbers of palliative patients and services provided.

The quantitative data analyses were conducted using SAS Enterprise Guide 7.1. (SAS Institute Inc, Cary, NC, USA) The spatial analyses and the maps were conducted and designed in ESRI^®^ArcGIS™ 10.7.1 Esri Inc., Redlands, CA, USA.

## 3. Results

### 3.1. Description of the Overall Sample

In total, 6940 patients in M-V received an initial palliative care assessment between 2014 and 2017 (Table 2), with 48.9% of the SAPV patients being female. The mean age was 73.3 years (95% CI: 73.0, 73.6). For 18.6% (*n* = 1293) of the patients, the SAPV teams had a travel distance of >30–50 km. For another 9.6% (*n* = 668), the distance was >50 km. The total number of treatment days was 245,611, with an average of 35.4 (95% CI: 34.1, 36.7) treatment days per patient.

### 3.2. Number of Patients, Treatment Days per Patient and Distance

Regarding the number of patients per 10,000 inhabitants, there is a trend of an increasing rate of patients with increasing distance to the treating SAPV team’s site (Table 2). This trend is not statistically significant. However, the average number of treatment days per patient in a distance of >50 km is significantly higher compared to the service areas ≤30 km and >30–50 km.

**Figure 1:**
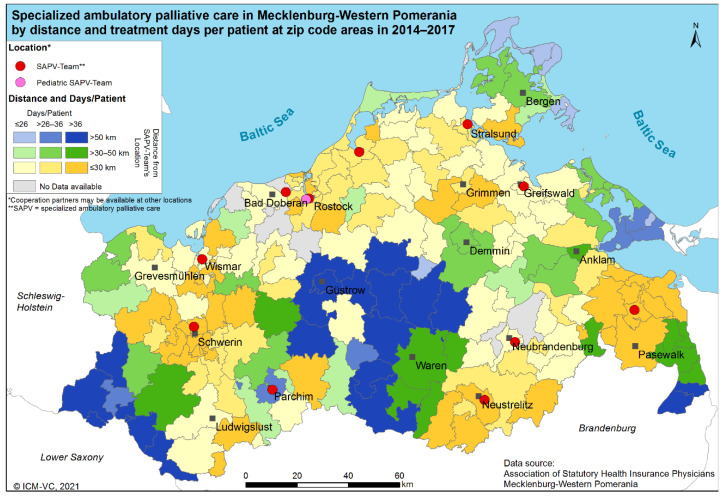
SAPV in Mecklenburg-Western Pomerania by distance and treatment days per patient at the level of zip code areas in 2014–2017. * Cooperation partners may be available at other locations. ** SAPV = specialised ambulatory palliative care.

Figure 1 shows the treatment days per patient by distance. The legend shows a colour matrix representing distance by hue and treatment days per patient by colour intensity. The matrix is read as follows: the darker the blue, the longer the distance and the more treatment days per patient, and the lighter the yellow, the shorter the distance and the less treatment days per patient.

If a patient’s zip code can be allocated to several distance categories in equal numbers, the lower distance category is selected in this case. This can lead to an underestimation of the distances. A map on the basis of the highest categories (overestimation of the distances) is pictured in the supplement (Figure S1).

For a detailed spatial analysis of the treatment days per patient, a general cluster method (Global Moran’s I) was used to identify a tendency towards spatial heterogeneity or clustering of the utilisation of SAPV services. The Global Moran’s I analysis showed a significant trend towards clustering (Moran’s Index = 0.29; z-value = 6.78; *p*-value < 0.01). This result was confirmed by the use of local Moran’s I indicators. There are high-high clusters over all distances, i.e., an agglomeration of neighbouring zip code areas with many treatment days per patient. Low-low clusters occur around the city of Bergen on the island Rugia and in the regions without data. Outliers that include a high-low or low-high relationship of neighbouring zip code areas regarding the number of treatment days per patient appear over all distances.

### 3.3. SAPV Services in a Distance of More Than 30 km

For SAPV patients who live more than 30 km away from the SAPV team, 81,429 treatment days were conducted in total. On 26.5% (*n* = 21,546) of these treatment days, the distance surcharge was actually reimbursed; thereof, 14,250 days in a distance of >30–50 km and 7296 days in a distance of >50 km (Table 3).

Overall, the average number of treatment days per patient without a distance surcharge is higher than with a surcharge (Table 3). In a distance of >50 km, the mean number of treatment days per patient without a distance surcharge is very high (41.2 days) compared to the mean number of treatment days with a distance surcharge (10.9 days) and shows a statistically significant difference to the less distant service area.

SAPV home visits comprise nursing and physician services (Table 3). If both days with and without a distance surcharge are included, more days with nursing home visits (*n* = 19,207) than with physician home visits (*n* = 9648) were conducted. In a distance of >50 km, no physician home visit was conducted with a distance surcharge, with significant difference to the less distant service area. This shows that cooperating physicians conduct a large part of specialised palliative home care in a distance of >50 km.

### 3.4. SAPV Utilisation over Time

Between 2014 and 2017, the total number of patients with SAPV treatment increased (Table 4). Up to a distance of 30 km, the number of patients increased from 1031 in 2014 to 1608 in 2017 (+56.0%). At a distance between >30 and 50 km, the number of patients increased from 229 in 2014 to 435 in 2017 (+90.0%). The largest increase in utilisation of SAPV is in patients living >50 km from the SAPV team’s site, from 95 in 2014 to 250 in 2017 (+163.2%). The average number of treatment days per patient increased nearly equally between 2014 and 2017 in all areas of distance. It is higher at a further distance than in less distant regions over all years.

## 4. Discussion

### 4.1. Relation between Number of Patients, Treatment Days per Patient and Distance to the SAPV Team’s Site

The analyses of SAPV treatment in M-V for the period 2014 to 2017 revealed regional differences. On average, patients in a distance of more than 50 km of the SAPV team’s site received more treatment days per patient than in less distant regions. A cause might be that there are less other treatment possibilities in distant regions. SAPV teams are often based in central places where there are other palliative-specific or alternative services (e.g., general practitioners with palliative care qualification, specialised nursing services, palliative care wards in hospitals, hospices, providers of mourning support) [18]. In these regions, palliative patients can access other palliative care, whereas patients further away are likely more dependent on SAPV [25]. Areas in which an SAPV team frequently reimburses for distance surcharges may also indicate a lack of regional cooperation partners (cooperating physicians or nurses). Patients in those regions may depend on the SAPV teams. However, the cluster analysis showed that there are concentrations of many treatment days per patient over all distances. This result suggests that also other parameters than distance have an influence on the utilisation of SAPV. Causes might be differences in socioeconomic status, the prescribing behaviour by primary care physicians or the degree of attention by nursing services.

Various studies analysed the association between geographical accessibility and the utilization of healthcare services. Chukwusa et al. reported that there are variations in travel time to inpatient palliative and end-of-life facilities between rural and urban areas in England, UK, and that geographical access to palliative services is a determinant for place of death [26]. Patients living in rural regions are less likely to die at inpatient palliative and end-of-life facilities than urban patients [26]. Kaasalainen et al. identified that in Ontario, Canada, travelling long distances to visit palliative patients in rural communities impeded the care provision of nurses [27]. Moreover, several studies proved that time and distance have an influence on medical care, although these geographical factors are not always decisive alone [28,29,30]. The results of all these studies indicate that geographical accessibility has an influence on the utilisation of healthcare services. The majority of studies focus on the patient’s accessibility to medical facilities such as hospitals, general practitioners and specialist physicians, but there is little research on providing ambulatory medical care to patients. However, there is an important difference. In contrast to other healthcare services, SAPV teams conduct home visits. Their patients are usually so severely ill that they are no longer mobile. Large distances involve high travel times that can exceed the capacity of the SAPV teams. In the consequence, fewer patients can be treated.

### 4.2. SAPV Services by Healthcare Providers and Distance to the SAPV Team’s Site

In a distance of more than 50 km, there are more treatment days per patient than in less distant regions. For most of these days, the daily lump sum was charged, which includes daily tasks outside the home visits and involves consulting services, e.g., phone calls. Home visits by cooperating partners accounted for a much smaller part of care. Raknes et al. found that in a Norwegian primary care clinic, telephone consultations increased slightly with an increasing distance and that face-to-face utilisation of out-of-hour services decreased with an increasing distance [31].

If a differentiation is made between home visits by type of healthcare provider (physician/nurse), differences between the service areas become apparent. In total, there are more home visits in less distant regions. This seems plausible, since each house visit involves time and for remote locations an enormous amount of time is needed [19,32]. This finding is in line with Goodridge et al. who found out that residents in rural Canadian locations receive less home care services, such as palliative care, than residents in urban settings [33]. However, our data show no differences in the mean number of home visits per patient between the service areas. The mean number of home visits without a distance surcharge show that in a distance >30 km, a large part of specialised palliative care is conducted by cooperating health service providers. Since in a distance up to 30 km no distance surcharge is billed, no distinction can be made between services by the SAPV team or cooperation partners.

Time may also be the reason, why from a distance of more than 50 km, the physician home visit has not been charged at all by a SAPV team itself. Physician home visits from this distance are apparently carried out exclusively by cooperating physicians located much closer to the patient’s residence. Due to the long distances and the time it takes, the SAPV teams could possibly take care of fewer patients. Cooperating partners in the regions can solve this problem to some extent. The importance of cooperation partners for a regionwide SAPV is stated by Schneider et al., who analysed structural and process effects of SAPV in Bavaria, Germany, with particular regard to rural areas [34]. The authors state that networking of different actors is crucial for the supply of palliative care, as it enables provision close to patients’ residencies and within a reasonable amount of time. The importance of networking and collaboration between the individual healthcare providers is also shown by Rehner at al. [35] in a cross-sectoral survey, which identified palliative and hospice care in M-V to be more accessible to patients from urban regions than to patients from rural areas. The authors identified problems regarding the interface between outpatient and inpatient palliative care and between general and specialised palliative care, and suggested, for example, coordination units or outpatient consultation services to strengthen the provision of palliative care in rural regions. To improve transitions between curative therapy, general and specialised ambulatory palliative care, and to establish palliative care regionwide, complementary palliative services named “specially qualified and coordinated palliative care” (BQKPMV) were established in October 2017. BQKPMV is conducted by primary care physicians or specialists with further education [36]. The extent to which BQKPMV contributes to the cooperation of service providers needs to be further investigated.

### 4.3. Utilisation of SAPV over Time

Up to a distance of 50 km, there was a continuous increase in treatment days per patient between 2014 and 2017. The increase could be due to an increase in the size and capacity of the SAPV teams or changes in the individual care of each patient, e.g., supply requirements or supplies intensity.

An additional SAPV team was established in the northwest of M-V in 2019. However, with currently 13 SAPV teams, there is presently no comprehensive population-based supply. In order to implement the assessment of one SAPV team per 100,000 inhabitants [5], 16 to 17 teams would be needed in M-V [18].

### 4.4. Strengths and Limitations

This study is limited due to missing data of two SAPV teams, which creates a possible bias in the respective areas. The low-low clusters of local Moran’s I are, therefore, of very limited value. The regional lack of data may also have contributed to the outliers (neighbouring areas with different values), which requires further research. Some patients who live close to an SAPV team could have been treated by a team further away. Given the distances between adjacent SAPV areas, the number of these patients will likely be small. Furthermore, no data on number, location and capacity of cooperation partners are available. Cooperative relationships as well as other palliative care service providers, e.g., basic ambulatory palliative care and inpatient facilities, need to be considered in order to evaluate regional capacities and compensation possibilities comprehensively [17,18,25]. Additionally, the reimbursement data do not allow to evaluate the quality of patient care, severity of disease, requirement for specialised palliative care and intensity of care. At the federal state’s borders edge effects may occur. Since zip code areas are not equal to administrative areas, some zip code areas cross the border of M-V. This may cause a bias in the population-based data. In addition, SAPV teams from other federal states can care for some patients in M-V whose data would then not be represented in the data used.

Nonetheless, the strength of this study is that data of all available ambulatory statutory health insurance patients in M-V could be analysed. Additionally, due to the availability of data at the five-digit zip code level, small-scale spatial analyses could be carried out.

## 5. Conclusions

The population-based analysis of specialised ambulatory palliative care enables the identification of regional disparities. The distance between the SAPV team’s site and the patient’s residential location has an influence on the utilisation of SAPV services. However, it is essential to consider the kind of supply. Regional differences in the SAPV are not only related to the distance between the patient’s place of residence and the SAPV team’s location. Local structures have an important impact on the regional supply patterns and the kind of supply must be considered in order to be able to draw convincing conclusions about the utilisation of SAPV. More research on these topics is needed, including data from all health sectors and health professionals involved in the delivery of palliative care in order to comprehensively analyse regional palliative care for patients in M-V.

## Figures and Tables

**Table 1:** Palliative-specific, specialised ambulatory services according to the Association of Statutory Health Insurance Physicians in Mecklenburg-Western Pomerania.

Code	Service
99100	Initial assessment
99101	Lump sum per treatment day
99102	Nursing home visit
99103	Physician home visit
99106	Distance surcharge for distance >30 km
99107	Nursing home visit >30–50 km
99108	Physician home visit >30–50 km
99109	Nursing home visit >50 km
99110	Physician home visit >50 km

**Table 2:** Number of SAPV patients and treatment days, differentiated by distance to the treating SAPV team’s site (2014–2017).

	Total	Patients in a Distance of ≤30 km	Patients in a Distance of >30–50 km	Patients in a Distance of >50 km
Number of patients, *n*	6940	4979	1293	668
Number of patients per 10,000 inhabitants Mean (95% CI)	47.3 (41.1, 53.4)	43.6 (35.2, 52.0)	52.7 (44.2, 61.2)	59.3 (47.9, 70.7)
Age (years) Mean (95% CI)	73.3 (73.0, 73.6)	73.6 (73.3, 74.0)	72.7 (72.1, 73,4)	72.3 (71.4, 73.2)
Female (%)	48.9	49.1	49.3	46.7
Treatment days, n	245,611	164,182	47,640	33,789
Treatment days per patient Mean (95% CI)	35.4 (34.1, 36.7)	33.0 (31.5, 34.5)	36.8 (34.0, 39.7)	50.6 * (45.5, 55.7)

* *p* < 0.05; as compared to the service areas ≤30 km and >30–50 km.

**Table 3:** Services with and without distance surcharge for patients in a distance of >30 km from the SAPV team’s site (2014–2017).

	>30–50 km		>50 km	
	With Distance Surcharge	Without Distance Surcharge	With Distance Surcharge	Without Distance Surcharge
**Total**				
Treatment days, *n*	14,250	33,390	7296	26,493
Number of patients, *n*	1293	1125	668	643
Treatment days per patient Mean (95% CI)	11.0 (10.3, 11.8)	29.7 * (27.1, 32.2)	10.9 (9.6, 12.2)	41.2 * (36.9, 45.5)
**Thereof**				
Days with nursing home visit, *n*	11,215	453	7296	243
Number of patients, *n*	1061	55	668	28
Days with nursing home visit per patient Mean (95% CI)	10.6 (9.8, 11.3)	8.2 (4.9, 11.5)	10.9 (9.6, 12.2)	8.7 (6.0, 11.4)
Days with physician home visit, *n*	6436	218	0	2994
Number of patients, *n*	1275	44	0	420
Days with physician home visit per patient Mean (95% CI)	5.0 * (4.7, 5.4)	5.0 (3.0, 6.9)	0 * (0, 0)	7.1 (6.2, 8.0)

* *p* < 0.05; comparing the categories >30–50 km and >50 km.

**Table 4:** Number of patients and treatment days per patient, by distance and year.

Distance to SAPV Team’s Site/Year	2014	2015	2016	2017
**Number of patients, *n***				
Total	1355	1735	2068	2293
≤30 km	1031	1234	1448	1608
>30–50 km	229	332	389	435
>50 km	95	169	231	250
**Treatment days per patient**				
Total Mean (95% CI)	28.3 (26.3, 30.3)	31.4 (29.4, 33.5)	33.0 (31.0, 35.0)	36.9 (34.8, 38.9) *
≤30 km Mean (95% CI)	26.7 (24.4, 29.0)	30.4 (28.0, 32.8)	30.5 (28.3, 32.8)	34.1 (31.8, 36.5) *
>30–50 km Mean (95% CI)	29.0 (24.4, 33.6)	32.6 (27.5, 37.7)	34.3 (29.9, 38.7)	38.7 (34.1, 43.3) *
>50 km Mean (95% CI)	43.7 (34.6, 52.8)	36.7 (28.9, 43.4)	46.2 (39.0, 53.3)	51.1 (43.3, 58.8)

* *p* < 0.05; as compared to 2014.

## Data Availability

Restrictions apply to the availability of these data. Data was obtained from the Association of Statutory Health Insurance Physicians in M-V (Germany) and are available from the authors with the permission of the Association of Statutory Health Insurance Physicians in M-V (Germany).

## Supplementary Materials

The following supporting information can be downloaded at: https://www.mdpi.com/article/10.3390/ijerph20032231/s1, Figure S1: SAPV in Mecklenburg-Western Pomerania by distance and treatment days per patient at the level of zip code areas in 2014–2017.
