# Supplementary figures and images for: Population-Based, Spatial Analysis of Specialised Ambulatory Palliative Care in Mecklenburg-Western Pomerania, Germany, on the Basis of Reimbursement Data
Source: Int J Environ Res Public Health. 2023 Jan 26;20(3):2231. doi: 10.3390/ijerph20032231 (PMC9916229; doi:10.3390/ijerph20032231)

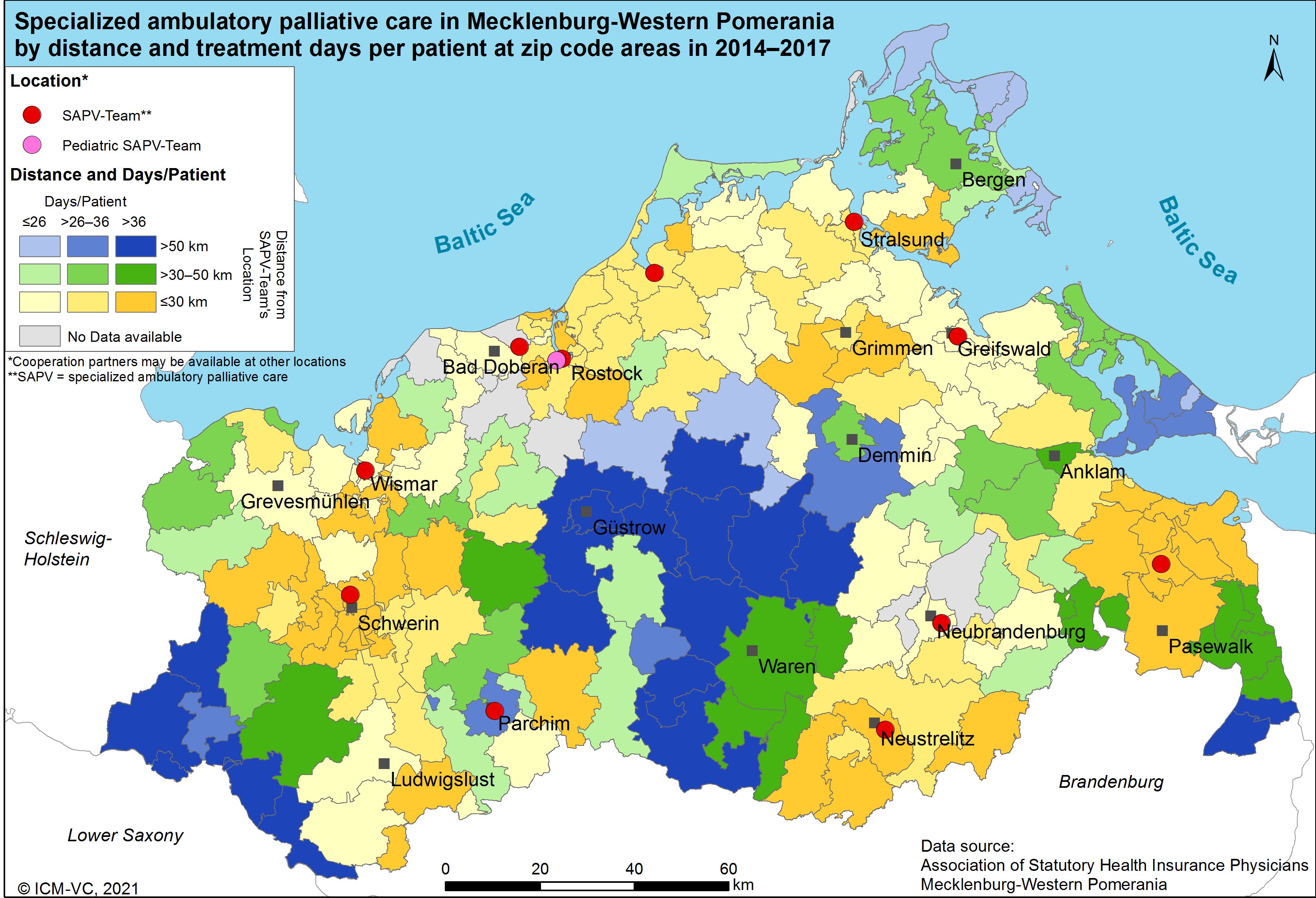

Supplement: Supplementary file 1 [file ijerph-20-02231-s001.zip › Figure S1.jpg]
